# Androgen Receptor Signaling Positively Regulates Monocytic Development

**DOI:** 10.3389/fimmu.2020.519383

**Published:** 2020-10-15

**Authors:** Camila Rosat Consiglio, Sandra O. Gollnick

**Affiliations:** ^1^Roswell Park Comprehensive Cancer Center, Department of Immunology, Buffalo, NY, United States; ^2^Roswell Park Comprehensive Cancer Center, Department of Cell Stress, Buffalo, NY, United States

**Keywords:** androgen receptor, enzalutamide, monocyte development, myelopoiesis, 5-FU (5-fluorouracil)

## Abstract

Myeloid cells are critical cells involved in the orchestration of innate and adaptive immune responses. Most myeloid cells derive from the adult bone marrow in a process called myelopoiesis, a tightly controlled process that ensures constant production of myeloid cells. Sex differences in myeloid cell development have been observed; males exhibit greater monocytic differentiation in the bone marrow, and men have increased blood monocyte numbers when compared to women. Here we use a genetic mouse model of myeloid androgen receptor (AR) knockout (MARKO) and pharmacological inhibition of AR to investigate the role of androgen signaling in monocytic differentiation. We observe that although myeloid AR signaling does not influence total bone marrow cell numbers, it does affect the composition of the bone marrow myeloid population in both homeostatic and emergency settings. Genetic deletion of AR in myeloid cells led to reduced monocytic development *in vivo*. Similarly, pharmacologic inhibition of AR signaling *in vitro* reduced monocytic development. However, alteration in monocytic differentiation in the absence of AR signaling did not lead to reduced numbers of circulating myeloid cells, although MARKO male mice display reduced ratio of classical to non-classical monocytes in the blood, implying that blood monocyte subsets are skewed upon myeloid AR deletion. Our results suggest that the sex differences observed in monocytic differentiation are partly attributed to the positive role of the androgen–AR axis in regulating monocytic development directly at the myeloid cell level. Furthermore, we have identified a novel role for AR in regulating blood mature monocyte subset turnover. Investigating how androgen signaling affects monocytic development and monocyte subset heterogeneity will advance our understanding of sex differences in monocytic function at homeostasis and disease and can ultimately impact future therapeutic design targeting monocytes in the clinic.

## Introduction

Myeloid cells are critical to tissue homeostasis and in the orchestration of innate and adaptive immune responses. Monocytes are a heterogeneous set of myeloid cells composed of different sizes and functions. Monocytes have been implicated in diseases such as atherosclerosis and cancer (1). Understanding the regulation of monocytic production in homeostatic and stress settings can ultimately lead to the development of therapies that modulate monocyte numbers and function in disease. Myeloid cell development, or myelopoiesis, is a tightly regulated process that occurs in the adult bone marrow (BM), to ensure constant production of myeloid cells throughout an individual’s life (2, 3). During monocytic development, monocytic growth factors, such as granulocyte-macrophage and macrophage colony-stimulating factors (GM- and M-CSF respectively), and specific transcription factors coordinate the development of myeloid progenitors in specific BM niches. The earliest myeloid progenitors, known as common myeloid progenitors (CMPs), give rise to granulocyte/monocyte progenitors (GMPs); and within the GMP population, a monocytic progenitor (MoP) subpopulation produces mature monocytes that exit the BM and circulate in the bloodstream (4, 5). Monocytes that enter the blood circulation are named classical monocytes (CMs). Most of CMs leave the circulation to replenish myeloid subpopulations in different organs, while a few remain in the blood and transition into intermediate monocytes (IMs), and subsequently into non-classical monocytes (NCMs) (6). Blood NCMs patrol vasculature to remove damaged cells or debris (6). At inflammatory sites, such as in the tumor microenvironment or infection sites, CMs differentiate into monocyte-derived macrophages or dendritic cells depending upon microenvironmental cues. A constant production of monocytes and the ability to upregulate monocytic cells upon stress are key and impact inflammation, disease progression, and resolution (7).

Sex differences have been observed in disease prevalence and outcome (8). Interestingly, sex differences in monocyte development have also been described and may be a mechanism involved in sex-biased disease progression. M-CSF is expressed at higher levels in the BM of male mice compared to female mice (9). In addition, male BM cells cultured in colony-forming assays show increased monocyte and monocyte–granulocyte colony-forming units (CFU-M and CFU-GM) numbers when compared to females, indicating increased monocytic differentiation (9). Replenishment of peritoneal macrophages from BM monocytes is increased in male *versus* female mice (10, 11). During inflammatory settings, such as obesity, male mice have increased proportions of myeloid cell progenitors when compared to females (12). During inflammation of the peritoneum, male mice show greater increases in blood classical monocytes when compared to females (13). In humans, men have higher proportion of blood monocytes when compared to women (14). The sex bias in monocytic development occurs in post-pubertal setting where fluctuations in sex hormones occur, suggesting a role for sex hormone signaling in the regulation of monocytic development. Specifically, a positive role for testosterone and androgen receptor (AR) signaling may explain the sex differences observed. Studies examining the effects of AR signaling in myeloid cell development provide initial hints into potential mechanisms. Global AR knockout (GARKO) mice display no changes in CMP progenitors (15), although there is an observed decrease in bone marrow macrophages in GARKO relative to WT mice (16). Moreover, G-, GM- or M-CSF treatment of GARKO or WT bone marrow cells does not induce changes in CFU-G, -GM or -M, suggesting AR expression in progenitors is not involved with GMP differentiation (15). Nonetheless, GARKO mice are neutropenic and show reduced percentage of monocytic cells in the blood as compared to WT mice (15, 16). However, it remains unclear how AR expression in either progenitors or mature myeloid cells affects myelopoiesis in homeostasis and under stress conditions.

In this study, we utilized a genetic mouse model in which AR expression in myeloid cells has been genetically deleted (MARKO) and pharmacological inhibition of AR to understand the role of AR on myeloid cell development under homeostasis and myeloablative stress. We show that AR positively regulates BM monocytic development and that myeloid cell AR deletion alters the BM myeloid compartment. We further identify that AR expression in myeloid cells impacts blood monocyte subsets, with MARKO male mice exhibiting decreased percentage of CM. Further, the rate of monocytic production is impaired in MARKO male mice under myeloablative conditions. Importantly, we identify that AR positively affects monocytic differentiation by impacting not only mature myeloid cells, but also by reducing monocytic differentiation at the progenitor level.

## Material and Methods

### Animal Studies

Seven to ten week-old C57BL/6 male mice were purchased from Taconic Laboratory (Hudson, NY); LysM^cre^ C57BL/6 male and B6.129(Cg)-Gt(ROSA)26Sor^tm4(ACTB-tdTomato,-EGFP)Luo^/J mice were purchased from The Jackson Laboratory (Bar Harbor, ME). AR^floxed^ mice were generated by De Gendt Lab at Katholieke Universiteit Leuven (17) and kindly shared by Agoulnik Lab at Florida International University. For the generation of Myeloid AR KnockOut (MARKO) mice, Lys-M^cre^ males were crossed with AR^floxed^ females to generate MARKO males. Control male mice for the experiment, referred to as WT mice, consisted of either AR^floxed^ littermates or syngeneic C57BL/6 male mice. Mice were housed in microisolator cages in a laminar flow unit under ambient light at 24°C. The RPCCC Institutional Animal Care and Use Committee (IACUC) approved all procedures and experiments for this study.

### *In Vivo* Experiments

For *in vivo* enzalutamide treatments, WT C57BL/6 male mice were treated daily with 100 μl of 20 mg/kg enzalutamide (Selleckchem S1250) by oral gavage or vehicle for 5 or 14 days. For emergency myelopoiesis experiments, WT and MARKO male mice were treated i.p. with 150 mg/kg 5-fluorouracil (5-FU, Invivogen sud-5fu) intraperitoneally and followed over time.

### Blood Analyses

Terminal blood collection was performed following CO_2_ euthanization of mice. Blood leukocyte populations were determined by complete blood count (CBC) analysis. For flow cytometric analyses of blood leukocytes, blood was collected, RBC lysed and stained with surface antibodies (below).

### Primary Cultures

Total bone marrow from C57BL/6 mice was initially lineage depleted by incubation with anti-mouse lineage antibodies (Anti-B220 eBioscience cat# 14-0452-82, anti-CD11b eBioscience cat# 14-0112-82, anti-Ter119 eBioscience cat# 14-5921-82) using magnetic beads (Qiagen cat# 310107). Lineage depleted bone marrow was then FACS-sorted for oligopotent GMP cells based on expression of specific markers (Sca-1^−^ c-Kit^+^ CD16/32^+^ CD150^-^ Ly6C^-^ CSF1R^-^). Sorted oligopotent GMPs were cultured in 10 ng/ml of IL-3 (Preprotech AF-213-13) and 10 ng/ml of SCF (Preprotech AF-250-03) in 200 μl of phenol red free RPMI 1640 (ThermoFisher 11835030) supplemented with 100 μg/ml Penicillin–Streptomycin–Glutamine (ThermoFisher 10378016) and 10% FBS Premium Select (Atlanta S11595) at 37°C and 5% CO_2_. Experiments were performed in the presence/absence of 5 μM enzalutamide (Selleckchem S1250) in 96 well plates at 1,000 cells/well. The concentration of enzalutamide utilized was determined previously (18), and was chosen based on concentrations used in the literature for *in vitro* cultures (19, 20). Experiments with M-CSF stimulation of oligopotent GMPs were performed in the same conditions in the presence of 10 ng/ml of M-CSF.

Bone marrow-derived macrophages (BMDMs) were generated by culturing 1 × 10^6^ unfractioned bone marrow cells from C57BL/6 male mice with 30 ng/ml M-CSF (ThermoFisher 14-8983-80) in the presence of DMSO or 5 μM enzalutamide (Selleckchem S1250) in 10 cm dishes in phenol red free RPMI 1640 (ThermoFisher 11835030) supplemented with 100 μg/ml Penicillin–Streptomycin–Glutamine (ThermoFisher 10378016) and 10% FBS Premium Select (Atlanta S11595) at 37°C and 5% CO_2_ for 5 days.

### Flow Cytometry

For flow cytometry staining, single cell suspensions were incubated with antibodies against cell surface molecules for 40 min on ice. Cells were then washed twice, and analysis was done at flow cytometer (BD Fortessa, BD LSRII). Analyses were performed using FlowJo™. Using FlowJo plugins (https://flowjo.com/exchange/#/), dimensionality reduction was performed by downsizing samples to 25 K cells each, concatenating samples and performing UMAP using the default settings (Euclidean distance, nearest neighbors of 15, and minimum distance of 0.5). Percentage of cre-expressing myeloid cells was determined by flow cytometry using LysM^cre^ ROSA^mT/mG^ mice. Antibodies used for flow cytometry were CD45 (BD 550994), CD11b (BD 553311), F4/80 (BioLegend 123149), CD115 (ThermoFisher 12-1152-82), Ly-6G (BioLegend 127612), Ly-6C (BioLegend 128033), CD16/32 (eBioscience 45-0161-82), Sca-1 (eBioscience 25-5981-82), CD150 (BioLegend 115927), c-Kit (BioLegend 105826), Ter-119 (eBioscience 48-5921-82), B220 (eBioscience 57-0452-82), CD4 (BioLegend 100427), CD8a (eBioscience 48-0081-82), CD3e (ThermoFisher MA5-17658), IRF8 (eBioscience 17-9852-82), PU.1 (Cell signaling 2216S), CD43 (BD 560663), and LD UV (ThermoFisher L23105).

### Statistical Analysis

Statistical analyses were performed using GraphPad Prism 8.0 software. When comparing two groups, statistical analyses were performed using two-tailed Mann–Whitney or paired tests. When comparing two groups or more groups, 1-way or 2-way ANOVA was performed. Multiple comparison correction (Bonferroni correction) was applied when necessary. Differences were considered significant when P values were ≤ 0.05.

## Results

### Androgen Receptor Is Implicated in Bone Marrow Monocytic Development

**Figure 1:**
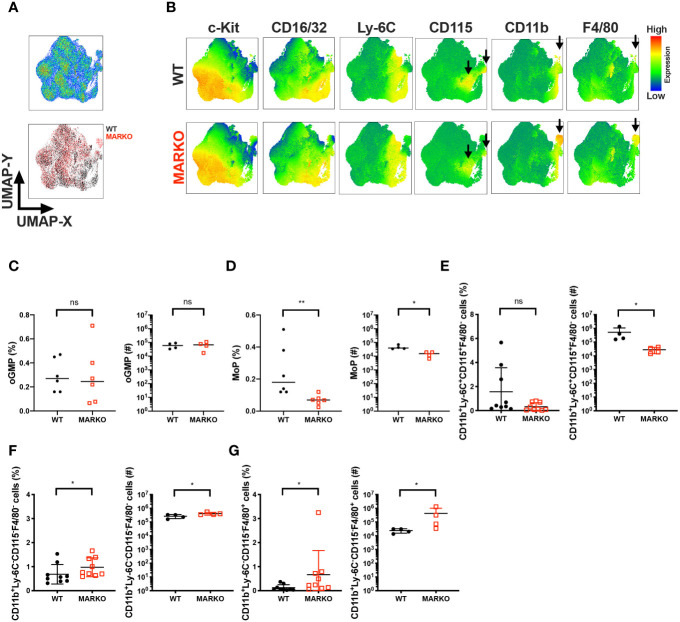
AR is important for bone marrow monocytic development. BM of WT and MARKO male mice was analyzed by flow cytometry for monocytic and macrophage cell populations. **(A)** The upper plot represents UMAP analysis of live singlet lineage negative (CD3, Ly-6G, B220, Ter119) BM cells from WT and MARKO male mice. The lower plot in **(A)** is colored according to sample group. **(B)** UMAP graphs indicating intensities of c-Kit, CD16/32, Ly-6C, CD115, CD11b and F4/80 expression in WT and MARKO BM. **(C–G)** Quantification of BM cell populations utilizing gating schemes in **Supplementary Figures 1A, D, F**. Plots depict percentage and numbers of singlet live **(C)** oligopotent GMP (oGMP), **(D)** monocytic progenitors (MoPs), **(E)** CD11b^+^Ly6C^+^CD115^+^F4/80^−^ cells, **(F)** CD11b^+^Ly6C^−^CD115^−^F4/80^−^ cells, and **(G)** CD11b^+^Ly6C^−^CD115^-^F4/80^+^ cells. Graphs show pooled data from two to four experiments with two to three mice per group. Black filled squares denote WT, and red empty squares indicate MARKO BM. Comparisons were done with non-parametric *t*-test. ns, not significant; *p ≤ 0.05, **p ≤ 0.01.

Sex differences are observed in monocyte development in the bone marrow and blood monocyte levels; males generally have increased levels of monocytes (9–14). To understand whether testosterone activation of AR signaling accounts for this sex difference and affects monocytic cell development in male mice, we generated Myeloid AR KnockOut (MARKO) mice, which lack AR expression in mature myeloid cells (**Supplementary Figure 1**). Bone marrow of WT and MARKO male mice was analyzed by flow cytometry using dimensionality reduction of single cells by uniform manifold approximation and projection (UMAP) algorithms (21). UMAP was performed on singlet live lineage negative cells (Lineage CD3, Ly-6G, B220, Ter119) to visualize clusters of developing myeloid cells and mature non-granulocytic myeloid cells in an unbiased manner. Cell clusters were identified using the parameters c-Kit, Sca-1, CD150, CD16/32, Ly-6C, CD115, CD11b and F4/80, with WT and MARKO male BM displaying differences in clusters (**Figure 1A**). To define the different populations, we analyzed the expression of myeloid markers within the UMAP coordinates. We identified differences between WT and MARKO BM, with MARKO BM displaying reduced CD115 expression within two populations, and increased expression of CD11b and F4/80 (**Figure 1B**, arrows indicate differences). To further dissect how AR expression impacts myeloid cell development, we analyzed developing myeloid cell populations by manual flow cytometry gating (**Supplementary Figure 2A**). WT and MARKO males did not display differences in total bone marrow cell numbers, nor in the percentage of granulocyte–monocyte progenitors (GMPs) (**Supplementary Figures 2B, C**). Further analysis of GMPs identified three subpopulations of GMPs: oligopotent GMP (oGMP), granulocytic progenitor (GP), and monocytic progenitor (MoP) populations (**Supplementary Figure 2D**). While AR deletion in myeloid cells did not affect oGMP and GP cell percentage and numbers (**Figure 1C** and **Supplementary Figure 2E**), MARKO male mice displayed significantly reduced MoP percentage and cell numbers (**Figure 1D**), suggesting a positive role for AR during monocytic development. To further understand whether AR impacts the mature non-granulocytic myeloid compartment in the BM, mature myeloid cell populations were identified by manual flow cytometry gating (**Supplementary Figure 2F**). WT and MARKO BM did not differ in mature CD11b^+^Ly-6C^−^CD115^+^F4/80^−^, CD11b^+^Ly-6C^+^CD115^−^F4/80^−^, or CD11b^+^Ly-6C^−^CD115^+^F4/80^+^ myeloid cell populations (**Supplementary Figures 2G–I**). However, BM isolated from MARKO male mice displayed reduced numbers of mature CD11b^+^Ly-6C^+^CD115^+^ BM monocytes when compared to BM from WT mice (**Figure 1E**). Moreover, MARKO BM displayed increased percentage and number of cells in the non-monocytic and non-granulocytic CD11b^+^Ly-6C^−^CD115^−^F4/80^−^ population (**Figure 1F**) and increased percentage and number of cells in the macrophage CD11b^+^Ly-6C^−^CD115^−^F4/80^+^ population (**Figure 1G**) when compared to WT BM, corroborating the differences visualized through UMAP (**Figure 1B**). These results suggest that AR expression significantly impacts the myeloid bone marrow compartment by increasing monocytic development.

### Androgen Receptor Impacts the Percentage of Blood Monocyte Subset

**Figure 2:**
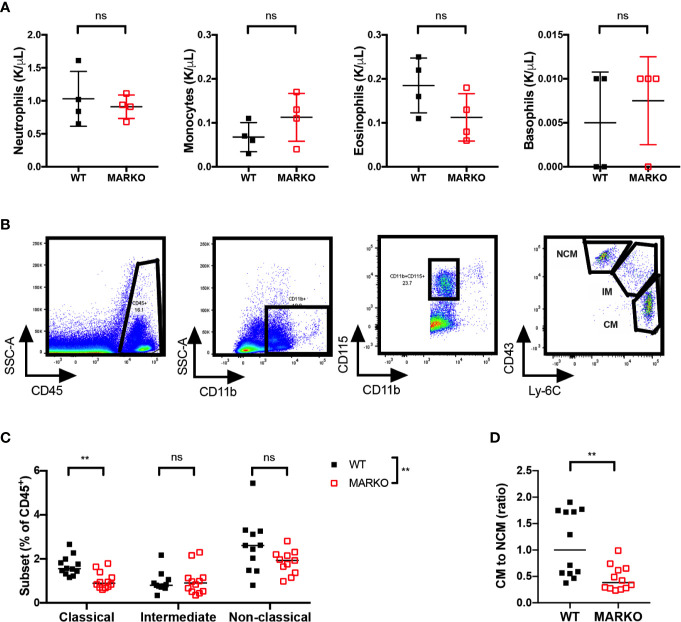
AR deletion decreases ratio of classical to non-classical blood monocytes. Blood leukocytes of WT and MARKO male mice were collected and assessed by complete blood count (CBC) and flow cytometry. Plots depict quantification of blood **(A)** neutrophils, monocytes, eosinophils, and basophils by CBC. **(B)** Flow cytometry gating strategy utilized to distinguish classical (CM), intermediate (IM), and non-classical (NCM) blood monocytes. **(C)** Monocyte subset percentage out of total CD45^+^ blood cells. **(D)** Ratio of the percentage of blood CM to NCM. Graphs show pooled data from two to four experiments with two to three mice per group. Black filled squares denote WT, and red empty squares indicate MARKO BM. Graph **(C)** was compared by two-way ANOVA, comparisons in **(A, D)** were done with non-parametric *t*-tests. ns, not significant; **p ≤ 0.01.

It is possible that the reduction in BM monocytes observed in MARKO mice impacts circulating blood monocyte numbers. To test this hypothesis, we analyzed blood leukocyte numbers of WT and MARKO male mice by complete blood counts (CBC). No differences were observed in total white blood cell (WBC) numbers (**Supplementary Figure 3A**), nor in the individual myeloid populations of neutrophils, monocytes, eosinophils, and basophils (**Figure 2A**); thus, AR deletion does not appear to affect blood monocyte numbers. To understand whether AR alters monocyte subsets, we quantified the percentage of CD11b^+^CD115^+^ monocyte subsets within leukocytes in WT and MARKO male blood by flow cytometry. Classical (CM), intermediate (IM), and non-classical (NCM) monocyte subsets were identified based on Ly-6C and CD43 expression (**Figure 2B**). Indeed, MARKO males exhibited altered percentage of monocyte subsets when compared to WT males, with a decreased percentage of CM and reduced ratio of the percentage of CM to NCM (**Figures 2C–D**). These results imply that AR positively regulates BM monocytic development and blood CM subset.

### The Rate of Monocytic Production Is Impaired in MARKO Male Mice

**Figure 3:**
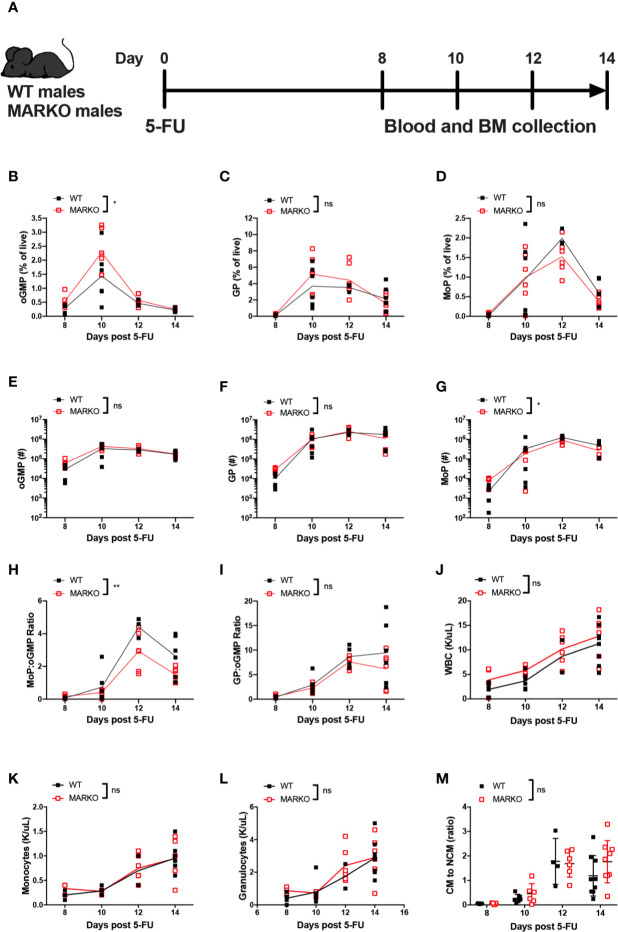
Deletion of myeloid cell AR is associated with reduced bone marrow monocytic progenitor production following 5-FU bone marrow ablation. **(A)** Experimental design: WT and MARKO male mice were injected intraperitoneally with 5-fluorouracil (5-FU), and BM and blood samples were collected 8, 10, 12 and 14 days following treatment. Plots depict percentage of singlet live BM **(B)** oligopotent GMPs (oGMPs), **(C)** granulocytic progenitors (GPs), and **(D)** monocytic progenitors (MoPs). Graphs indicate total number of BM **(E)** oGMP, **(F)** GP, and **(G)** MoP. Graphs denote ratio of the percentage of BM subpopulation **(H)** MoP to oGMP, and **(I)** GP to oGMP. **(J–L)** CBC quantification of **(J)** total white blood cells (WBCs), **(K)** blood monocytes, and **(L)** blood granulocytes. **(M)** Ratio of blood classical monocytes (CM) to non-classical monocytes (NCMs). Graph shows data from two to three experiments with two mice/group/time point. Black filled squares denote WT, and red empty squares indicate MARKO samples. Statistical analyses were done by two-way ANOVA. ns, not significant; *p ≤ 0.05, **p ≤ 0.01.

To understand how myeloid AR affects the rate of monocytic production, mice were exposed to myeloablative therapy by intraperitoneal injection with a single sublethal dose of 5 fluorouracil (5-FU). Recovery of BM and blood cells was analyzed 8, 10, 12, and 14 days after 5-FU injection (**Figure 3A**). Myeloablative therapy induced weight loss in both WT and MARKO mice; MARKO males showed small but significant increase in weight 14 days after 5-FU injection when compared to WT males (**Supplementary Figure 3B**). No differences in recovery of total BM cell count were observed (**Supplementary Figure 3C**). GMP production peaked at day 10 following 5-FU for both WT and MARKO mice, and percentage of GMPs were similar between WT and MARKO mice (**Supplementary Figure 3D**). BM isolated from MARKO mice displayed significantly increased percentage of oGMP, and no changes in the percentage of GP and MoP were produced; however, MARKO BM MoP numbers showed a significant reduction following 5-FU when compared to WT mice (**Figures 3B–G**). These results suggest that rather than faster production and early release of monocytes into the bloodstream, MARKO mice fail to produce as many monocytic progenitors as WT mice following myeloablative therapy. Indeed, the ratio of MoP to oGMP production, but not the ratio of the percentage of GP to oGMP, is reduced in MARKO mice when compared to WT mice, indicating a specific impairment in the monocytic differentiation pathway (**Figures 3H, I**). Nonetheless, the reduced rate of monocytic production in MARKO does not affect recovery of blood WBC, granulocyte or monocyte numbers following 5-FU (**Figures 3J–L**). No differences were observed in the percentage of blood CM, IM, and NCM, nor in the ratio of CM to NCM during the 14 days of recovery (**Figure 3M** and **Supplementary Figures 3E–G**).

### Pharmacological AR Antagonism Reduces Monocytic Differentiation at the Progenitor Level

**Figure 4:**
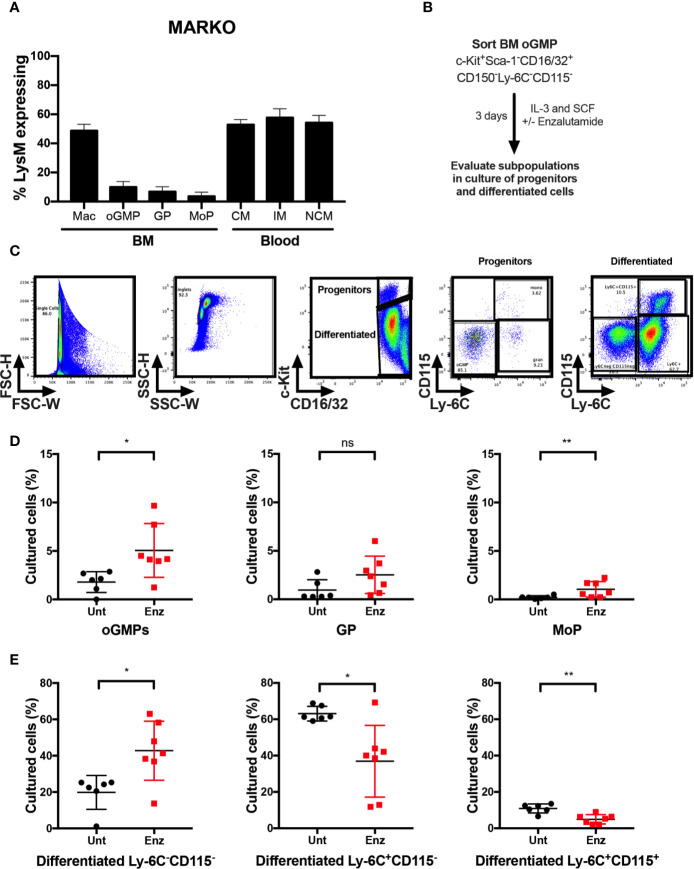
AR antagonism delays monocytic development *in vitro* in the absence of differentiating signals. **(A)** Bone marrow and blood of LysM^cre^ ROSA^mT/mG^ mice were collected and percentage of cre-expressing myeloid cells was analyzed by flow cytometry to evaluate MARKO model penetrance. **(B)** Experimental design: Oligopotent GMP (oGMP) population was sorted from WT male BM and cultured *in vitro* with stem cell factors IL-3 and SCF in the presence or absence of enzalutamide for three days and differentiation was assessed by flow cytometry. **(C)** Flow cytometry gating of untreated and enzalutamide-treated cultured cells on day 3. **(D, E)** Graphs depict percentage of **(D)** progenitor oGMP, granulocytic progenitors (GPs), monocytic progenitors (MoPs) and **(E)** differentiated cell populations in cultures at day 3. Graphs show data of three experiments with two biological replicates each. Black filled squares denote WT untreated, and red filled squares indicate enzalutamide-treated samples. Comparisons were done by non-parametric *t*-test. ns, not significant; *p ≤ 0.05, **p ≤ 0.01.

To identify which cell types contributed to the changes in monocytic development observed in MARKO males, blood and BM from LysM^cre^ ROSA^mT/mG^ mice were collected, and percentage of cre-expressing myeloid cells was analyzed by flow cytometry to evaluate MARKO model penetrance of AR deletion during myelopoiesis. AR deletion was observed in about half of BM macrophages, identified as bulk CD11b^+^F4/80^+^ cells. (**Figure 4A**). Further, BM progenitor populations of oGMP, GP and MoP had very low penetrance of AR deletion (~5–10% of progenitor populations), while 50–60% of blood monocyte subsets lacked AR (**Figure 4A**). These results suggest that the effects observed in MARKO male mice occur as monocytes are differentiating, and it is likely that mature BM myeloid cells are influencing the monocytic differentiation, as has been previously demonstrated (22–24). To test the hypothesis that AR signaling can also impact monocytic differentiation at the progenitor level, BM oGMP cells were sorted from WT males, cultured in media containing stem cell factors IL-3 and SCF in the presence or absence of the second-generation AR antagonist enzalutamide; differentiation was assessed after 3 days of culture by flow cytometry (**Figure 4B** and **Supplementary Figures 4A–B**). Differentiation of oGMP was quantified based on c-Kit, CD115, and Ly-6C expression (**Figure 4C**). After 3 days of culture, the majority of cells in culture differentiated in both groups, as the percentage of c-Kit^−^ cells was higher than c-Kit^+^ (**Figures 4D, E**). AR antagonism resulted in delayed differentiation, as the percentage of progenitor cells (oGMP and MoP) was significantly higher in enzalutamide-treated when compared to untreated samples (**Figure 4D**). Enzalutamide skewed the differentiation of oGMPs towards non-monocytic c-Kit^−^ cells, as the percentage of Ly-6C^−^CD115^−^ cells increased while the percentage of both Ly-6C^+^CD115^−^ and Ly-6C^+^ CD115^+^ cells decreased in enzalutamide-treated when compared to untreated cultures (**Figure 4E**). These results suggest that in the absence of a monocytic differentiating factor, AR inhibition blocks monocytic differentiation at the progenitor level and results in increased non-monocytic differentiation.

**Figure 5:**
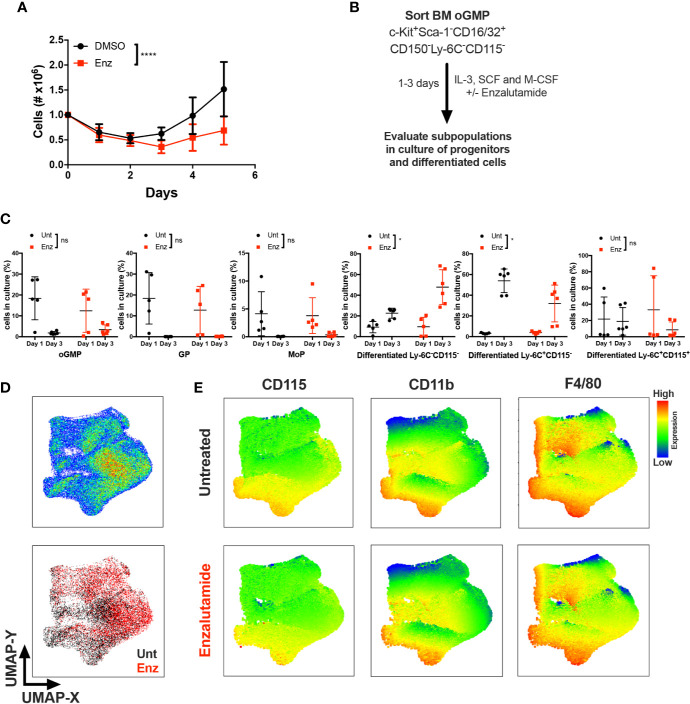
AR antagonism delays monocytic development *in vitro* in the presence of differentiating M-CSF signal. **(A)** Unfractioned WT bone marrow cells were cultured *in vitro* with M-CSF in the presence of DMSO or enzalutamide (Enz) for 5 days. Graph depicts **(A)** cell numbers after 1, 2, 3, 4, and 5 days of culture. **(B)** Experimental design, where oligopotent GMP (oGMP) population was sorted from WT male BM, cultured *in vitro* with IL-3, SCF, and M-CSF, and left untreated (Unt) or treated with enzalutamide for one and three days. Differentiation was assessed by flow cytometry. **(C)** Using gating strategy from **Figure 4C**, graphs depict percentage of oGMP, granulocytic progenitors (GPs), monocytic progenitors (MoPs) and differentiated cell populations in cultures at days 1 and 3. **(D)** The upper plot represents UMAP analysis of cultures at day 3 gated at singlet CD16/32^+^ population. The lower plot in **(D)** is colored according to sample group. **(E)** UMAP graphs indicate intensities of CD115, CD11b, and F4/80 expression in untreated and enzalutamide-treated cultures at day 3. Graphs show data of three experiments with one to two biological replicates/group each. Black filled squares denote WT untreated, and red filled squares indicate enzalutamide-treated samples. Statistical analyses in **(A, C)** were done by two-way ANOVA. ns, not significant; *p ≤ 0.05, ****p ≤ 0.0001.

Monocytic differentiation in the bone marrow is transcriptionally regulated and requires coordination of specific monocytic growth factors, such as GM-CSF, M-CSF and IL-34 (25–27). To test whether AR antagonism impacted monocytic differentiation in the presence of a monocytic growth factor, unfractioned WT BM cells were cultured *in vitro* with M-CSF in the presence of enzalutamide or DMSO for 5 days, and viable cell numbers were assessed. AR antagonism significantly reduced the number of monocyte/macrophage cells over 5 days of culture (**Figure 5A**). To further delineate the effect of AR antagonism on progenitors in the presence of a differentiating signal, BM oGMPs were sorted from WT males, cultured in media containing IL-3, SCF, and M-CSF in the presence or absence of enzalutamide, and differentiation was assessed after 1 and 3 days of culture by flow cytometry (**Figure 5B**; sorting strategy in **Supplementary Figures 4A, B**, and gating strategy for cultured cells in **Figure 4C**). oGMP cells cultured in the presence of M-CSF displayed increased differentiation over time, with the percentage of c-Kit^+^ cells decreasing in both groups from days 1 to 3 of culture (**Figure 5C**). Enzalutamide treatment did not result in differences from untreated samples in the percentage of progenitor populations over time. However, the percentage of differentiated non-monocytic Ly-6C^−^CD115^−^ cells significantly increased over time in enzalutamide-treated samples when compared to untreated even in the presence of a monocytic differentiating signal (**Figure 5C**). Enzalutamide treatment also reduced percentage of differentiated Ly-6C^+^CD115^−^ cells over time when compared to untreated (**Figure 5C**). In addition, the upregulation of the M-CSF receptor CD115 and the mature myeloid and macrophage markers CD11b and F4/80 were significantly reduced over time upon AR blockade as compared to control cultures (**Supplementary Figure 4C**). To visualize the effect of AR antagonism in monocytic differentiation *in vitro*, UMAP plots were generated utilizing singlet CD16/32^+^ cells after 3 days of culture in the presence of M-CSF. Unsupervised analysis revealed differential proportion of clusters between untreated and enzalutamide-treated cultures (**Figure 5D**). Enzalutamide-treated cultures displayed clusters with reduced CD115, CD11b, F4/80, and Ly-6C expression and no changes in c-Kit and CD16/32 (**Figure 5E** and **Supplementary Figure 4D**). Altogether these results suggest that AR antagonism skews progenitor differentiation away from monocytic development even in the presence of a monocytic differentiating signal.

## Discussion

**Figure 6:**
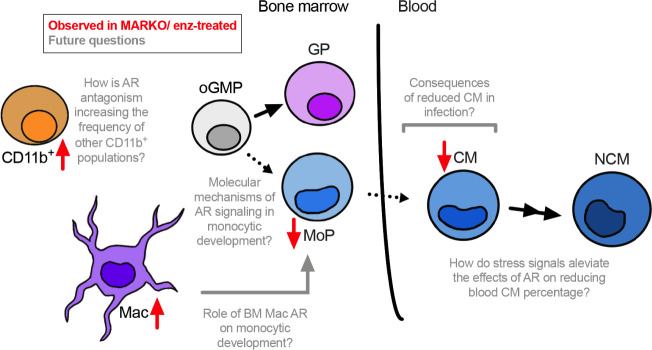
Overall model. AR positively regulates monocytic development. Through *in vitro* studies using enzalutamide (enz) and *in vivo* experiments using myeloid AR knockout (MARKO) male mice, we demonstrate that lack of AR is involved with reduced monocytic development in the bone marrow and increased presence of mature BM cells, such as macrophages (Mac) and non-granulocytic non-monocytic CD11b^+^ cells. In addition, reduced monocytic development is accompanied by changes in blood monocyte subsets, where myeloid AR deletion reduced the percentage of classical monocytes (CMs). Several questions can now be pursued in terms of molecular mechanisms by which AR impacts monocytic development and its consequences during inflammatory or infectious conditions.

Understanding how sex hormone receptors impact myelopoiesis is key to dissecting mechanisms involved in monocytic function in health and disease. Here we demonstrate that AR blockade at both the progenitor and mature myeloid cell level leads to changes in BM composition, and results in reduced monocytic differentiation in male mice (**Figure 6**).

Using a murine model with AR-deficient myeloid cells, we observe a reduction in different CD115^+^ monocytic cell populations, from monocytic progenitors to mature BM monocytes. Reduced ratio of MoP to oGMP but no increases in GP to oGMP ratio following myeloablation with 5-FU suggests that AR deletion can block monocytic differentiation rather than skew GMPs towards granulocytic differentiation. Similarly, oGMP differentiation into MoP was diminished in the presence of a pharmacological AR inhibitor *in vitro* when M-CSF was both present and absent. Our results imply that AR is important for monocytic differentiation, and lack of AR signaling reduces monocytic differentiation. In addition, enzalutamide treatment decreases monocyte/macrophage cell numbers when bulk bone marrow containing HSCs is cultured with M-CSF. These results suggest that not only differentiation, but also proliferation and/or survival is impacted by AR expression in myeloid cells. The reduced numbers of cultured cells could be a consequence of increased cell death or slower proliferation. In prostate cells, AR expression is associated with cell survival and proliferation. In prostate cancer, androgens can stimulate cell cycle progression by AR-mediated regulation of G1–S transition, while androgen ablation triggers cell death and cell cycle arrest (28). Conversely, AR knockout in B cells is associated with increased proliferation and decreased apoptosis of these cells (29), while AR knockout in neutrophils reduces the proliferative capacity of neutrophils and retards their maturation (15). Our findings relate to the previous study, where we observe delayed monocytic maturation in MARKO males. Therefore, it is possible that AR might be involved not only in differentiation, but also in the proliferative capacity of monocytic progenitors. However, AR does not appear to regulate recovery following 5FU-induced emergency myelopoiesis.

As monocytic differentiation was reduced in MARKO males under homeostatic and stress conditions, we hypothesized that this would result in reduced blood monocyte levels. Yet, MARKO male mice displayed normal monocyte blood counts; the lower classical to non-classical blood monocyte ratio in MARKO male mice implies that AR signaling affects developing monocytes as well as mature monocyte subsets. Nonetheless, monocyte subset ratio is not affected up to 14 days following 5-FU. These results suggest stress signals may induce compensatory mechanisms that alleviate effects of AR on blood monocytes under stress conditions. Alternatively, the change in monocytic subsets in homeostasis can imply differential monocytic subset lifespan, migration or differentiation. Recently, we have shown that myeloid AR does not affect myeloid cell infiltration into subcutaneously implanted C2 prostate tumors, suggesting that AR does not affect blood monocytes tumor migration (18). Alveolar macrophages from MARKO male mice display decreased cytokine and chemokine production in a model of allergic lung inflammation, and these changes are associated with decreased eosinophil infiltration to lung (30). It still remains to be determined how AR is impacting monocyte subsets in models of infection and inflammation, and how decreased CM to NCM ratio impacts disease susceptibility and progression. As men have higher proportion of blood monocytes when compared to women, and monocyte proportions increase in women with age (14, 31), future studies should compare blood monocytes between sexes across lifespan to determine how sex hormone variation impacts these subsets.

We show that MARKO male mice have higher BM non-granulocytic non-monocytic CD11b^+^ cells and BM macrophages concomitantly with the reduction in BM monocytic differentiation. These results bring about two potential hypotheses: AR deletion skews differentiation of progenitor cells towards other non-monocytic myeloid cell types, and/or AR differentially impacts the survival of different myeloid population(s). Our *in vitro* experiments indicate that direct AR antagonism of GMPs leads to increased percentages of non-monocytic non-granulocytic CD11b^+^ cell in cultures even in the presence of M-CSF, indicating that AR acts directly on progenitors to skew their differentiation. More studies will be needed to specifically assess how GMP differentiation is being altered and which lineages are increasing with enzalutamide treatment, such as DC, basophil, and eosinophil lineages. Thus, it remains to be determined how AR expression in mature myeloid cells affects monocytic cell numbers in the BM. AR expression in mature myeloid cells may affect the bone marrow microenvironment to alter monocytic differentiation. M-CSF is expressed at higher levels in male BM compared to female (9), and it is possible that AR positively regulates M-CSF expression. Another factor involved in the activation of CSF-1R is IL-34, which has not been assessed in this study and could be involved with the reduced monocytic number observed. In addition, BM macrophages have been shown to maintain HSC niches (22, 23), while bone marrow myeloid cells are closely associated with developing myeloid cells and affect their quiescence and self-renewal (24). It is therefore possible that AR deletion in mature myeloid cells impacts monocytic development by disrupting/altering the communication between mature and developing myeloid cells.

By using a myeloid-specific knockout model, we were able to determine the specific contribution of myeloid AR to monocytic development. Some of our results contrast to previous work using a global AR knockout model, where decreased bone marrow macrophages and reduced percentage of blood monocytes were observed in GARKO mice when compared to WT (15, 32). This discrepancy could be a result of i) different penetrance of AR deletion in myeloid cells between the models, and/or ii) additive or compensatory effects of AR deletion in non-myeloid cells of GARKO mice. As androgens affect B cell development through modulation of androgen-sensitive BM stromal cells (33), it is possible that stromal cell AR also impacts monocytic development. Future studies should address the effect of AR expression by different cell populations on monocytic development to tease out how individual contribution of each component impacts the overall effect.

Overall, our results indicate that AR is an important player in the process of monocytic development and may explain some sex differences observed in monocyte biology. Furthermore, our results imply that monocytic development may be affected by androgen levels and therapies that aim on reducing androgen signaling, such as AR antagonists used in prostate cancer treatment. Understanding how sex hormones and sex hormone receptors regulate monocytic production in homeostatic and stress settings may ultimately aid in the development of therapies that modulate monocyte numbers and function in disease.

## Data Availability Statement

The datasets generated for this study are available on request to the corresponding author.

## Ethics Statement

The animal study was reviewed and approved by The Roswell Park Comprehensive Cancer Center (RPCCC) Institutional Animal Care and Use Committee (IACUC) approved all procedures and experiments for this study.

## Author Contributions

Conceptualization: CC and SG. Methodology: CC. Investigation and data analysis: CC and SG. Writing of original draft: CC and SG. Editing of original draft: CC and SG. Funding acquisition: SG. Supervision: SG. All authors contributed to the article and approved the submitted version.

## Funding

Research reported in this publication was supported in part by the National Cancer Institute of the National Institute of Health under Award 5P01CA98156 (SG) and the Roswell Park Alliance Foundation. The study used shared resources supported by Roswell Park Cancer Institute Cancer Center Support Grant (P30CA016056).

## Conflict of Interest

The authors declare that the research was conducted in the absence of any commercial or financial relationships that could be construed as a potential conflict of interest.
