# Supplementary material for: Androgen Receptor Signaling Positively Regulates Monocytic Development
Source: Front Immunol. 2020 Oct 15;11:519383. doi: 10.3389/fimmu.2020.519383 (PMC7604537; doi:10.3389/fimmu.2020.519383)
Supplement: Supplementary file 1 [file Presentation_1.pdf]

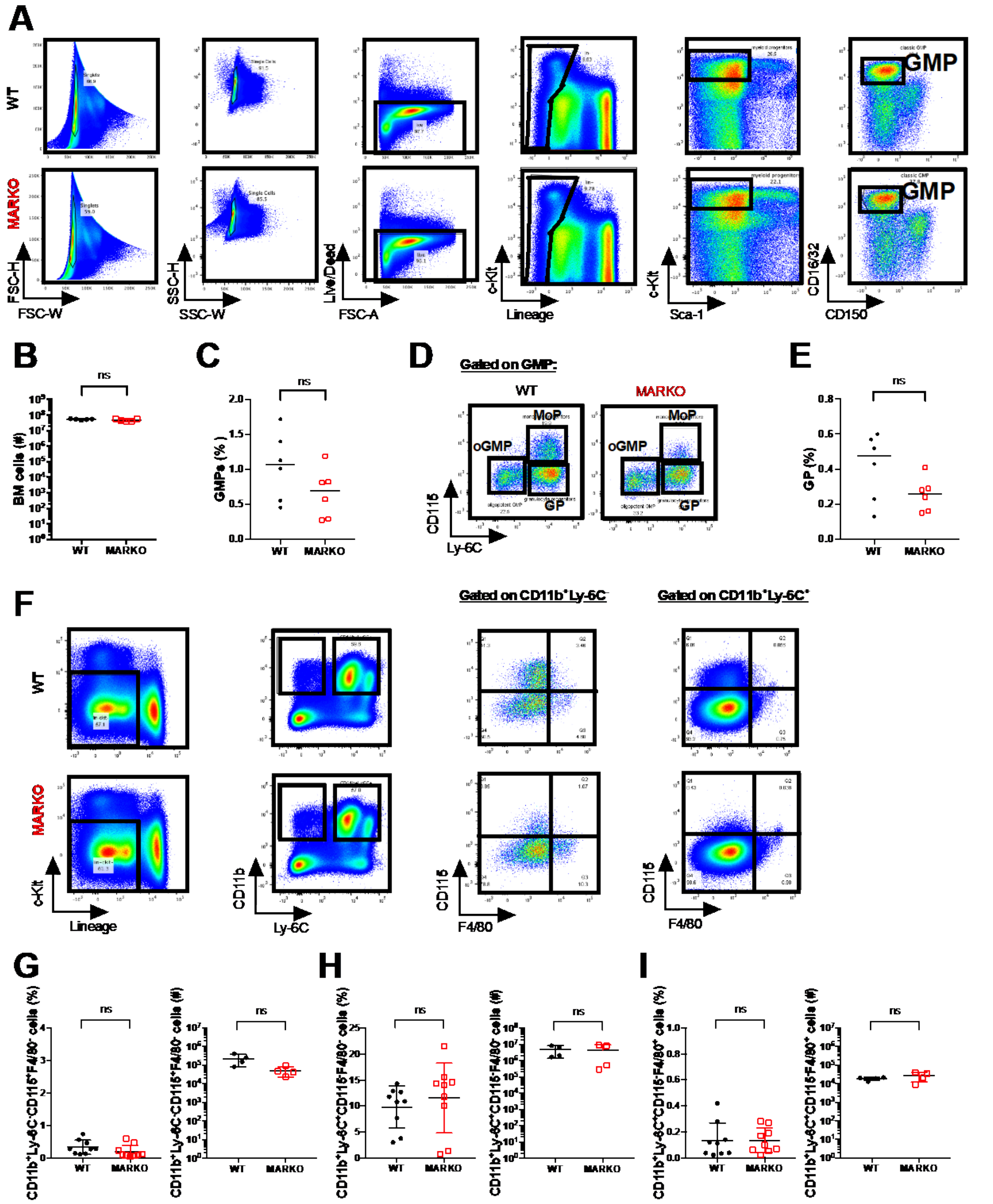

Supplementary Figure 2 AR is important for bone marrow monocytic development. **(A)** Flow cytometry gating strategy utilized for gating bone marrow (BM) granulocyte-monocyte progenitors (GMP) progenitor populations of WT and MARKO male mice. Lineage gating is based on CD3, Ly-6G, B220, and Ter119 expression. **(B)** Total BM cell numbers from WT and MARKO male mice. **(C)** Percentage of GMP within singlet live BM cells. **(D)** Subgating of GMP population into three subpopulations: oligopotent GMPs (oGMP), monocytic progenitors (MoP) and granulocytic progenitors (GP). (**E**) Percentage of GPs within singlet live BM cells. **(F)** Flow cytometry gating strategy utilized for gating of mature BM myeloid populations of WT and MARKO mice. (**G-I**) Graphs depict percentage within BM live singlets

and total numbers of **(G)** CD11b^{+}Ly6C^{-}CD115^{+}F4/80^{-} cells, **(H)** CD11b^{+}Ly6C^{+}CD115^{-}F4/80^{-} cells, and **(I)** CD11b^{+}Ly6C^{+}CD115^{-}F4/80^{+} cells. Graphs show pooled data from 2-4 experiments with 2-3 mice per group. Black filled squares denote WT, and red empty squares indicate MARKO BM. Comparisons were done with non-parametric t-test. ns=not significant.

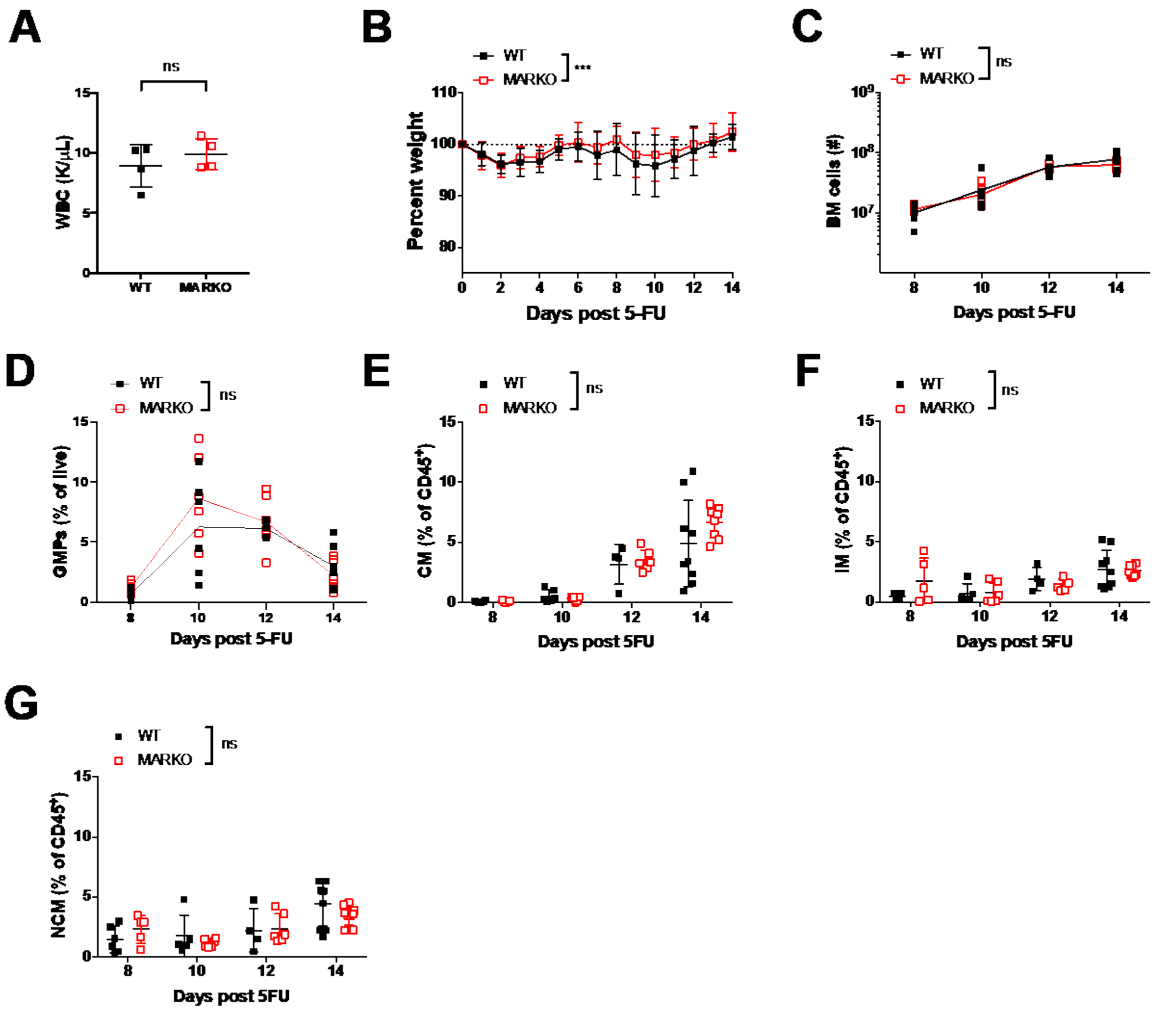

Supplementary Figure 3 Deletion of myeloid cell AR is associated with reduced bone marrow monocytic progenitor production following 5-FU bone marrow ablation. **(A)** Total white blood cell (WBC) quantification by complete blood counts (CBC). **(B-G)** WT and MARKO male mice were injected intraperitoneally with 5-fluorouracil (5-FU), and BM and blood samples were collected 8-, 10-, 12- and 14-days following treatment. **(B)** Percentage of initial weight of WT and MARKO mice following 5-FU treatment. **(C)** Total BM cell quantification following 8-, 10-, 12- and 14-days of 5-FU treatment. Graph depicts percentage of **(D)** granulocyte-monocyte progenitors (GMP) within live singlets following 5-FU treatment. Plots indicate percentage of blood **(E)** classical monocytes (CM), **(F)** intermediate monocytes (IM), and **(G)** non-classical monocytes (NCM) within CD45^{+} cells. Graph shows data from 2-3 experiments with 2 mice/ group/ time point. Black filled squares denote WT, and red empty squares indicate MARKO samples. Statistical analysis in **(A)** was done by non-

parametric t-test; analyses in **(B-G)** were done by two-way ANOVA. ns=not significant, ***$p \leq 0.001$.
